# Nucleus Pulposus Resorption Following a Paraspinal Hook‐Needle Intervention for Lumbar Disc Herniation: A Case Report

**DOI:** 10.1002/ccr3.73226

**Published:** 2026-07-23

**Authors:** Mengying Xiao, Zhengchao Cai, Hairui Zhu, Shuanglin Mou, Wensheng Zhu

**Affiliations:** ^1^ Health Science Center Hubei Minzu University Enshi China; ^2^ Department of Osteology Huanggang Hospital of Traditional Chinese Medicine Huanggang China

**Keywords:** case report, lumbar disc herniation, minimally invasive therapy, paraspinal hook‐needle intervention

## Abstract

The paraspinal hook‐needle intervention is a minimally invasive needle‐based procedure performed under local anesthesia using a specialized hook‐shaped needle at clinically selected lumbar segments. We report the case of a 70‐year‐old woman who presented with intermittent low back pain for more than 10 years, recently aggravated and accompanied by pain and numbness in the left lower limb for two weeks. Lumbar magnetic resonance imaging revealed disc herniation at L4/5 and L5/S1 with compression of the left L5 nerve root, along with a nodular intracanalar lesion at the L4/5 level suggestive of a sequestered nucleus pulposus. Following the paraspinal hook‐needle intervention, the patient experienced marked relief of low back pain and radicular symptoms, with restoration of normal gait. Physical examination demonstrated significant improvement in lumbar range of motion and substantial reduction in paraspinal muscle tension and tenderness. Muscle strength of ankle dorsiflexion and the extensor hallucis longus recovered to grade V, and sensory deficits in the L4–S1 dermatomes were markedly improved. The straight‐leg raise test improved to 70° without significant radicular pain. The visual analog scale score decreased from 7 to 3, and the Oswestry Disability Index score decreased from 57% to 23%. No recurrence or adverse events were observed during the 1‐year follow‐up period. This case documents clinical improvement after a paraspinal hook‐needle intervention and subsequent radiographic disappearance of sequestered nucleus pulposus. Nevertheless, spontaneous resorption is a recognized feature of the natural history of lumbar disc herniation and cannot be excluded in this patient. Therefore, the observed temporal association should not be interpreted as evidence of causality or established efficacy, and prospective controlled studies are needed to evaluate the safety and potential clinical role of this procedure.

## Key Clinical Message

In this single case, paraspinal hook‐needle intervention was temporally associated with clinical and neurological improvement and 1‐year radiographic disappearance of a sequestered nucleus pulposus fragment. Spontaneous resorption cannot be excluded, and efficacy, causality, and safety require evaluation in prospective comparative studies.

## 1 Introduction

Lumbar disc herniation (LDH) is a common cause of low back pain and radicular symptoms in the lower limbs [1]. It occurs when the annulus fibrosus becomes lax or ruptures, allowing the nucleus pulposus to protrude posteriorly and compress the spinal cord or nerve root [2]. Migration of herniated disc material within the spinal canal is a recognized manifestation of lumbar disc herniation, particularly in cases of extrusion or sequestration. Migrated or sequestered disc fragments may contain nucleus pulposus, annulus fibrosus, cartilaginous endplate tissue, or a combination of these components. Magnetic resonance imaging (MRI) provides a comprehensive assessment of the degree and location of sagittal disc protrusion, offering high diagnostic value for this condition [3].

Management of lumbar disc herniation is individualized according to symptom severity, neurological findings, imaging characteristics, duration of symptoms, and patient preference. In the absence of cauda equina syndrome or progressive neurological deterioration, initial management commonly includes activity modification, medication, rehabilitation, and other nonoperative measures [4]. Minimally invasive or surgical decompression may be considered when symptoms remain disabling, neurological deficits progress, or conservative management fails [5, 6]. Importantly, radiographic regression of lumbar disc herniation has been documented during nonoperative follow‐up, indicating that improvement in symptoms or imaging findings cannot automatically be attributed to a specific intervention [7]. A growing range of nonpharmacological and minimally invasive strategies has also been investigated in lumbar spine care; however, outcomes vary according to the clinical population and intervention, highlighting the need for rigorous comparative evaluation rather than extrapolation across different procedures [8].

Among minimally invasive needle‐based techniques [9], a specialized procedure using a hook‐shaped needle has been developed from concepts related to the traditional “New Nine Needles” and contemporary paraspinal soft‐tissue release techniques. In the present report, we use the term “paraspinal hook‐needle intervention” to describe this procedure and to distinguish it from manual spinal manipulation. The procedure involves the insertion and controlled manipulation of a specialized hook‐shaped needle within selected paraspinal soft tissues under local anesthesia. It does not involve manual mobilization of the spinal joints and is not intended to enter the intervertebral foramen, spinal canal, or epidural space [10]. The paraspinal hook‐needle intervention involves controlled stimulation and release of selected paraspinal soft tissues using a specialized hook‐shaped needle [11]. The paraspinal hook‐needle intervention is an integrative needle‐based procedure that combines biomedical segmental localization with a traditional paraspinal point framework. The clinically relevant spinal level is identified primarily by correlating the patient's symptoms and neurological findings with MRI [12] evidence of disc pathology and neural compression. Traditional terminology, including Wei's Jiaji point regions, is subsequently used to describe the corresponding bilateral paraspinal surface locations and does not replace anatomical or imaging‐based assessment. Site selection follows a hierarchical clinical process. First, the potentially responsible lumbar segment is identified by integrating the distribution of pain and sensory symptoms, neurological examination findings, and MRI abnormalities. Second, palpation is used within the clinically implicated paraspinal region to identify focal tenderness, increased muscle tension, or other soft‐tissue abnormalities and thereby refine the superficial intervention sites. The intervention is not routinely performed at spinal levels without relevant clinical, neurological, imaging, or local examination findings. Needle direction and depth are determined by the intended paraspinal tissue plane and individual anatomical characteristics rather than by meridian theory alone.

The paraspinal hook‐needle intervention is intended to release selected paraspinal soft tissues without directly removing the herniated disc fragment. However, evidence regarding its clinical effects as a standalone intervention remains limited. We therefore report the clinical course and 1‐year imaging findings of a patient with lumbar disc herniation and a sequestered fragment who underwent this procedure. This case is presented as a descriptive clinical observation rather than evidence that the intervention caused disc resorption or has established therapeutic efficacy.

## 2 Case History/Examination

### 2.1 Case History

Patient: A 70‐year‐old female presented to Huanggang Hospital of Traditional Chinese Medicine on January 27, 2024, with a history of intermittent low back pain for over 10 years, recently aggravated and accompanied by pain and numbness in the left lower limb for the past two weeks. Ten years prior, she experienced dull, localized low back pain, primarily in the lumbar region, which was tender to touch but relieved by bed rest. Two weeks before presentation, her low back pain worsened and was associated with left lower limb pain, predominantly affecting the posterior left thigh and lateral calf, with numbness on the lateral aspect of the left calf and the first three toes of the left foot. She had a prior history of left foot sprain but denied other trauma or systemic diseases. She reported that conservative treatments, including oral traditional Chinese medicine and topical herbal plasters, failed to provide effective or sustained symptom relief, significantly impacting her daily life.

### 2.2 Examination

Physical examination: The patient adopted a protective posture and walked cautiously, with markedly restricted lumbar range of motion. Lumbar flexion, extension, and left lateral bending elicited exacerbation of low back pain and increased radiating pain in the left lower limb. Significant tenderness was noted over the left L4–S1 intervertebral discs, accompanied by increased paraspinal muscle tone on the left side. No obvious scoliosis or structural spinal deformities were observed.

Neurological examination: Muscle strength was assessed using the Medical Research Council (MRC) scale, revealing grade IV strength in the quadriceps, dorsiflexors, extensor hallucis longus, and plantarflexors on the affected side. Sensory testing demonstrated mild hypoesthesia in the corresponding dermatomes, predominantly within the L4–S1 regions. Deep tendon reflexes were diminished at both the patellar and Achilles tendons. No pathological reflexes were elicited.

Nerve root tension tests: On the affected side, the straight‐leg raise test elicited radiating pain in the lower limb at 60°, indicating a positive result. The contralateral straight‐leg raise test was also positive. No saddle anesthesia or bladder/bowel dysfunction was observed.

Functional assessment: The visual analog scale (VAS) score was 7, and the Oswestry Disability Index (ODI) was 57%, indicating significant pain and functional impairment.

## 3 Differential Diagnosis, Investigations and Treatment

### 3.1 Differential Diagnosis

Based on the patient's clinical manifestations of chronic low back pain accompanied by unilateral lower limb pain and numbness, together with imaging findings, the following differential diagnoses were considered:

1. Lumbar spinal stenosis—commonly presents with low back pain and lower extremity neurological symptoms in elderly patients; however, this patient lacked typical neurogenic intermittent claudication
2. Piriformis syndrome—may cause radiating pain in the lower limb, but lumbar MRI demonstrated definite nerve root compression caused by lumbar disc herniation.
3. Degenerative lumbar instability—can lead to chronic low back pain, although no significant vertebral instability was observed on imaging examinations.
4. Cauda equina syndrome—was excluded because the patient had no bowel or bladder dysfunction, saddle anesthesia, or progressive bilateral lower limb weakness
5. Intraspinal tumor or infection—the nodular lesion observed in the spinal canal was considered in the differential diagnosis; however, MRI findings and clinical manifestations were more consistent with a sequestered nucleus pulposus.

### 3.2 Investigations

Lumbar MRI (Figure 1A): (1) Degenerative changes of the lumbar spine, with endplate inflammation at L4 and L5 and intervertebral disc degeneration; (2) disc herniation at L4/5 and L5/S1 with compression of the left L5 nerve root; along with a nodular intracanalar lesion at the L4/5 level suggestive of a sequestered nucleus pulposus. Based on imaging findings and clinical history, a diagnosis of L4/L5 lumbar disc herniation causing left L5 nerve root compression and subsequent numbness and pain in the left lower limb was made.

**FIGURE 1:**
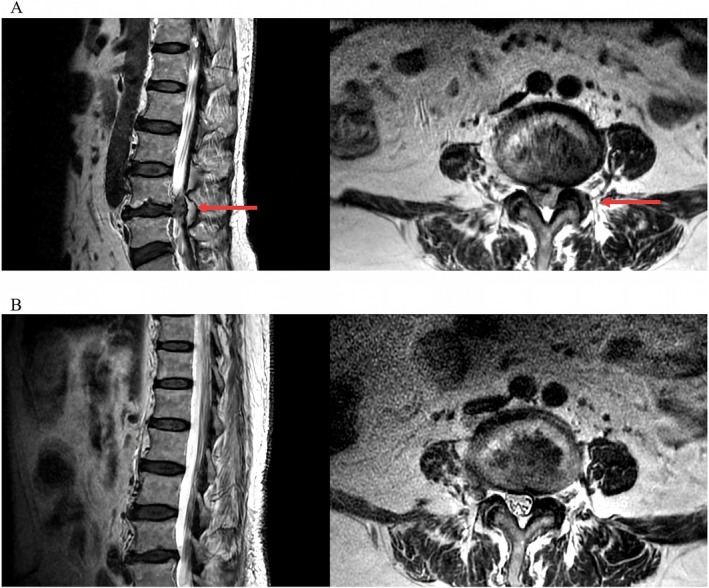
(A) Pre‐treatment lumbar magnetic resonance imaging (MRI) demonstrating L4–L5 disc herniation with inferior migration of the nucleus pulposus into the spinal canal. (B) Follow‐up lumbar MRI showing a patent spinal canal at the L4/5 level without evidence of residual sequestered nucleus pulposus.

### 3.3 Treatment

Upon admission, routine laboratory and diagnostic tests, including complete blood count, liver and renal function tests, coagulation profile, a standard panel for infectious diseases, cardiac enzyme spectrum, and electrocardiography, were performed to identify potential contraindications to the paraspinal hook‐needle intervention. After written informed consent was obtained, the patient underwent a paraspinal hook‐needle intervention on January 31, 2024. The intervention region was selected by correlating the patient's left‐sided radicular symptoms and neurological findings with the MRI abnormalities at L4/5 and L5/S1 and the evidence of left L5 nerve‐root compression. Palpation was then used to identify focal paraspinal tenderness and increased soft‐tissue tension within the clinically implicated lower lumbar region. The corresponding bilateral paraspinal sites were described using the traditional term “Wei's Jiaji point regions”; however, the selection of these sites was primarily based on the clinical and imaging findings rather than on meridian theory alone.

### 3.4 Procedure (Video S1)

The patient was positioned prone on the procedure table, with the lumbar region fully exposed. A 5‐cm soft cushion was placed beneath the umbilicus, and the face and nose aligned with the bed's circular aperture to allow unobstructed breathing. A specialized hook‐shaped needle (Figure 2) was used for the paraspinal hook‐needle intervention. Based on the correlation among the MRI findings, neurological examination, symptom distribution, and local paraspinal palpation, four superficial intervention sites were selected: approximately 2 cm lateral to the L4 spinous process bilaterally and approximately 2 cm lateral to the L5 spinous process bilaterally. (Figure 3A). The points were marked with a sterile pen and disinfected routinely. Local infiltration anesthesia was performed using 16 mL of 0.5% lidocaine hydrochloride, administering 4 mL per point. A disposable fenestrated drape was applied. The left hand held a sterile gauze to stabilize the tissue, while the right hand manipulated a sterilized, lumbar‐shaped hook needle. The curved tip was inserted along the skin, lifting and advancing sequentially through subcutaneous tissue, superficial fascia, and erector spinae muscle using controlled lifting and releasing movements, as demonstrated in Video S1 (Figure 3B). The procedure was terminated at the intended tissue plane when the operator perceived a reduction in soft‐tissue resistance. The needle depth was 2.5 cm. Upon withdrawal, slight pressure was applied around the puncture site to allow minimal bleeding (~2 mL) (Figure 3C). The skin flaps were restored, and a 2.0 × 2.0 g sterile cotton ball was applied to each point and secured, followed by application of a desensitizing adhesive dressing with pressure for 5 min. The procedure was completed smoothly. After the procedure, the patient was instructed to remain strictly supine for 12 h and underwent 24‐h cardiac monitoring. Vital signs remained stable throughout, with no adverse symptoms reported.

**FIGURE 2:**
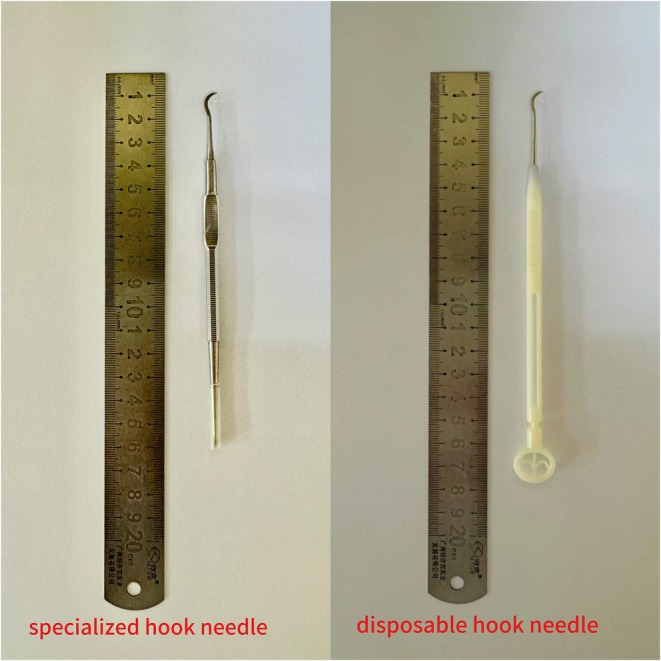
Specialized hook needle and disposable hook needle used for the paraspinal hook‐needle intervention.

**FIGURE 3:**
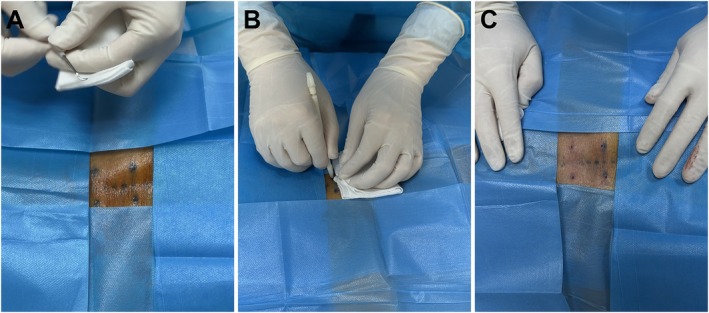
Procedural images of the paraspinal hook‐needle intervention: (A) Surface localization and marking of the selected paraspinal intervention sites, approximately 2 cm lateral to the L4 and L5 spinous processes bilaterally. (B) Controlled insertion and manipulation of the specialized hook‐shaped needle through the subcutaneous tissue, superficial fascia, and erector spinae muscle. (C) Mild bleeding at the puncture site after needle withdrawal, followed by local compression and dressing.

## 4 Conclusion and Results

One‐month post‐procedure follow‐up: At one month after the procedure, the patient reported further alleviation of low back pain and numbness in the left lower limb compared with the time of discharge. The visual analog scale (VAS) score decreased from 3 to 2, and the Oswestry Disability Index (ODI) decreased from 23% to 20%, reflecting notable improvement in daily functional ability. Lumbar range of motion had increased compared with prior assessments, and no significant radiating pain was observed.

Three‐month post‐procedure follow‐up: At three months after the procedure, the patient was able to walk normally and perform daily activities, experiencing only mild low back discomfort after prolonged sitting or exertion. Neurological examination showed maintained grade V strength in dorsiflexion and extensor hallucis longus muscles, and the straight‐leg raise test was negative. The Oswestry Disability Index (ODI) further decreased to 18%.

Six‐month post‐procedure follow‐up: At six months after the procedure, the patient's symptoms remained stable, with no recurrence of pain. Lumbar range of motion had largely returned to normal, and no sensory deficits or muscle weakness were observed.

Throughout the one‐year follow‐up, no adverse events such as infection, neurological injury, or symptom exacerbation were identified. The patient did not receive additional acupuncture, manual therapy, physical therapy, epidural injection, surgery, or regular analgesic medication during this period. She was advised to avoid excessive lumbar loading during the early recovery period and subsequently resumed ordinary daily activities gradually. Therefore, no major additional therapeutic intervention was documented that could otherwise explain the subsequent clinical course, although the natural history of lumbar disc herniation remains an important potential explanation.

One‐year post‐procedure follow‐up: Lumbar MRI (Figure 1B) performed at the outpatient clinic one year after the procedure revealed disc bulging from L2 to S1, with a patent spinal canal at the L4/5 segment and no evidence of sequestered nucleus pulposus. The patient reported no recurrence of low back or left lower limb pain and numbness, and neurological examination was normal.

## 5 Discussion

Lumbar disc herniation (LDH) is a common degenerative spinal disorder, primarily manifesting as low back pain and radicular symptoms, significantly affecting patients' functional status and quality of life. For patients with a disease duration of less than six months and without progressive neurological deficits, conservative treatment is typically recommended, with surgery reserved for cases refractory to conservative management [13, 14, 15]. In this case, the patient was elderly, and conservative therapy was considered unlikely to be effective; moreover, surgical intervention entails prolonged recovery, substantial economic burden, and the patient had high expectations for postoperative function and quality of life. Therefore, a paraspinal hook‐needle intervention was performed as a minimally invasive needle‐based procedure within the patient's treatment course. The clinical improvement observed after the paraspinal hook‐needle intervention represents a temporal association in this individual patient and should not be interpreted as evidence of established efficacy or as proof that the procedure can replace surgical or standard conservative treatment.

However, the effectiveness of the paraspinal hook‐needle intervention has not been established. A range of conservative, minimally invasive, surgical, and neuromodulatory strategies has been used for lumbar spine disorders, but evidence derived from one intervention or clinical population cannot be directly extrapolated to another. For example, the systematic review by Penchev et al. evaluated invasive and non‐invasive neuromodulation for pain after lumbar spine surgery and reported pain reduction without clear improvement in functional disability, highlighting the importance of assessing pain, function, safety, and long‐term outcomes separately [8]. However, these findings do not provide direct evidence for the paraspinal hook‐needle intervention used in the present case, and evidence supporting this procedure as a standalone treatment for lumbar disc herniation remains limited.

In this case, improvements in low back pain, radicular symptoms, neurological findings, and functional scores were observed after the paraspinal hook‐needle intervention. At the 1‐year follow‐up, MRI no longer demonstrated the previously observed sequestered nucleus pulposus. These observations demonstrate a temporal sequence between the intervention, clinical improvement, and subsequent radiographic change; however, they do not establish that the intervention caused either the symptomatic improvement or the resorption of the sequestered nucleus pulposus. Symptoms of lumbar disc herniation may fluctuate naturally over time, and clinical recovery does not necessarily occur in parallel with changes on MRI. Therefore, these findings should be interpreted as a descriptive observation from a single patient rather than evidence of treatment efficacy or causality.

Several mechanisms may be hypothesized to explain the early symptomatic improvement observed after the paraspinal hook‐needle intervention; however, these mechanisms were not directly evaluated in the present case and should therefore be interpreted cautiously.

First, the procedure may produce a local mechanical release effect by reducing paraspinal soft‐tissue tension and disrupting adhesions within the fascia and musculature [16]. Stimulation with the specialized hook‐shaped needle may also influence paraspinal muscle function, segmental mobility, and the mechanical properties of the surrounding fascial tissues [17, 18]. These local changes could potentially contribute to reduced mechanical irritation and improved lumbar mobility.

Second, stimulation of the paraspinal regions corresponding to Wei's Jiaji points may influence segmental sensory processing and neuromuscular function [19, 20]. Previous experimental and clinical studies of related acupuncture‐based interventions have suggested possible effects on neural modulation and pain signaling [21]. Nevertheless, direct evidence that the present procedure promotes axonal regeneration or restores neural function is lacking. The controlled lifting, mobilization, and release of selected paraspinal soft tissues may instead contribute to symptom relief through local mechanical and neuromodulatory effects.

Third, related acupuncture and acupotomy studies have proposed that local stimulation may affect tissue perfusion, inflammatory signaling, and nociceptive processing [22, 23, 24]. These mechanisms may provide a theoretical explanation for the early reduction in pain and radicular symptoms. However, the available evidence is indirect, and these biological effects were not measured in this patient. Therefore, they should not be regarded as confirmed mechanisms of the paraspinal hook‐needle intervention.

Spontaneous resorption should be considered separately from the possible mechanisms of early symptom improvement. Spontaneous regression of lumbar disc herniation is a recognized phenomenon [25, 26], particularly in extruded or sequestered disc material exposed to the epidural vascular and immune environment. Proposed mechanisms include dehydration and shrinkage of the fragment, retraction of herniated material, neovascularization, inflammatory degradation, and macrophage‐mediated phagocytosis. Substantial or complete radiographic regression during nonoperative follow‐up has been reported previously, including by Ilyov et al. [7]. Because the intervention in the present case was confined to the paraspinal soft tissues and did not directly enter, remove, or manipulate the sequestered nucleus pulposus, there is insufficient biological or clinical evidence to attribute the 1‐year MRI change to the procedure. The radiographic disappearance may therefore represent spontaneous resorption as part of the natural history of lumbar disc herniation.

The relatively rapid improvement in pain and neurological findings and the later disappearance of the fragment should also be interpreted as distinct observations occurring over different time scales. Early symptom improvement might be related to changes in local soft‐tissue tension, pain modulation, reduced inflammation, natural fluctuation, placebo or contextual effects, or a combination of these factors. In contrast, radiographic resorption generally develops over a longer period. Because no interval MRI examinations were performed between baseline and the 1‐year assessment, the timing and trajectory of fragment resorption are unknown.

These interpretive uncertainties also highlight several important limitations of this case report. First, this patient had a sequestered lumbar disc lesion, which represents a specific morphological subtype of lumbar disc herniation. Therefore, the findings from this patient cannot be generalized to patients with other morphological presentations, such as disc bulging, contained protrusion, or extrusion. It remains unknown whether the paraspinal hook‐needle intervention would be associated with similar clinical outcomes in these populations.

Second, although sequestered disc material may cause severe radicular pain, sensory disturbance, muscle weakness, or other neurological manifestations, it also has a considerable potential for spontaneous resorption. Accordingly, the clinical improvement and radiographic disappearance observed at the 1‐year follow‐up may reflect the natural evolution of the condition. Because this report lacks an untreated or standard‐conservative‐care control group and no interval MRI examinations were performed between baseline and 1 year, it is not possible to determine whether the observed clinical and radiographic outcomes were attributable to the intervention or to spontaneous recovery.

Third, the potential safety risks of the procedure should not be overlooked. In the present case, the intervention sites were located approximately 2 cm lateral to the L4 and L5 spinous processes, and the needle was advanced to a depth of approximately 2.5 cm through the subcutaneous tissue, superficial fascia, and erector spinae muscle. The intended trajectory was confined to the paraspinal soft tissues and was not directed toward the intervertebral foramen, spinal canal, or epidural space. This relatively superficial trajectory may reduce the likelihood of direct injury to the nerve root or dura mater compared with deeper transforaminal or epidural procedures; however, it does not eliminate these risks. Excessive insertion depth, an incorrect needle angle, anatomical variation, or unintended advancement could theoretically result in nerve‐root injury, dural puncture, cerebrospinal fluid leakage, neurological deterioration, vascular injury, bleeding, hematoma, or infection.

For these reasons, the procedure should be performed only by practitioners with detailed knowledge of lumbar anatomy and appropriate procedural training. Careful review of preprocedural imaging, accurate localization of the intended tissue plane, strict control of needle direction and depth, aseptic technique, and periprocedural neurological monitoring are essential. Image guidance may also warrant consideration in future standardized protocols, particularly when deeper structures are approached or anatomical landmarks are uncertain. Importantly, the absence of complications in this single patient does not establish the safety of the procedure. Future prospective studies should systematically document procedural details and adverse events and should include appropriate comparison groups before conclusions regarding safety or effectiveness can be drawn.

## Author Contributions

**Mengying Xiao:** writing – original draft, investigation, conceptualization. **Shuanglin Mou:** writing – review and editing, supervision, methodology. **Hairui Zhu:** conceptualization, investigation, writing – original draft. **Zhengchao Cai:** conceptualization, investigation, writing – original draft. **Wensheng Zhu:** methodology, writing – review and editing, funding acquisition, supervision.

## Funding

This work was supported by Research Fund Project of Huanggang Science and Technology Bureau (Grant JGYB20250011).

## Ethics Statement

This study was approved by the Biomedical Research Ethics Committee of Affiliated Huanggang Hospital of Traditional Chinese Medicine.

## Consent

Written informed consent was obtained from the patient for publication of this case report and accompanying images. The patient provided consent for the use of clinical information and imaging data for academic publication purposes.

## Conflicts of Interest

The authors declare no conflicts of interest.

## Supporting information


**Video S1:** Demonstration of the paraspinal hook‐needle intervention. The video demonstrates the controlled insertion, lifting, and releasing movements of the specialized hook‐shaped needle within the selected paraspinal soft‐tissue region. The video is intended to illustrate the procedural technique and does not indicate direct entry into the intervertebral foramen, spinal canal, epidural space, nerve root, dura mater, or sequestered nucleus pulposus.

## Data Availability

Research data are not shared.
