# Supplementary figures and images for: Nucleus Pulposus Resorption Following a Paraspinal Hook‐Needle Intervention for Lumbar Disc Herniation: A Case Report
Source: Clin Case Rep. 2026 Jul 23;14(8):e73226. doi: 10.1002/ccr3.73226 (PMC13392623; doi:10.1002/ccr3.73226)

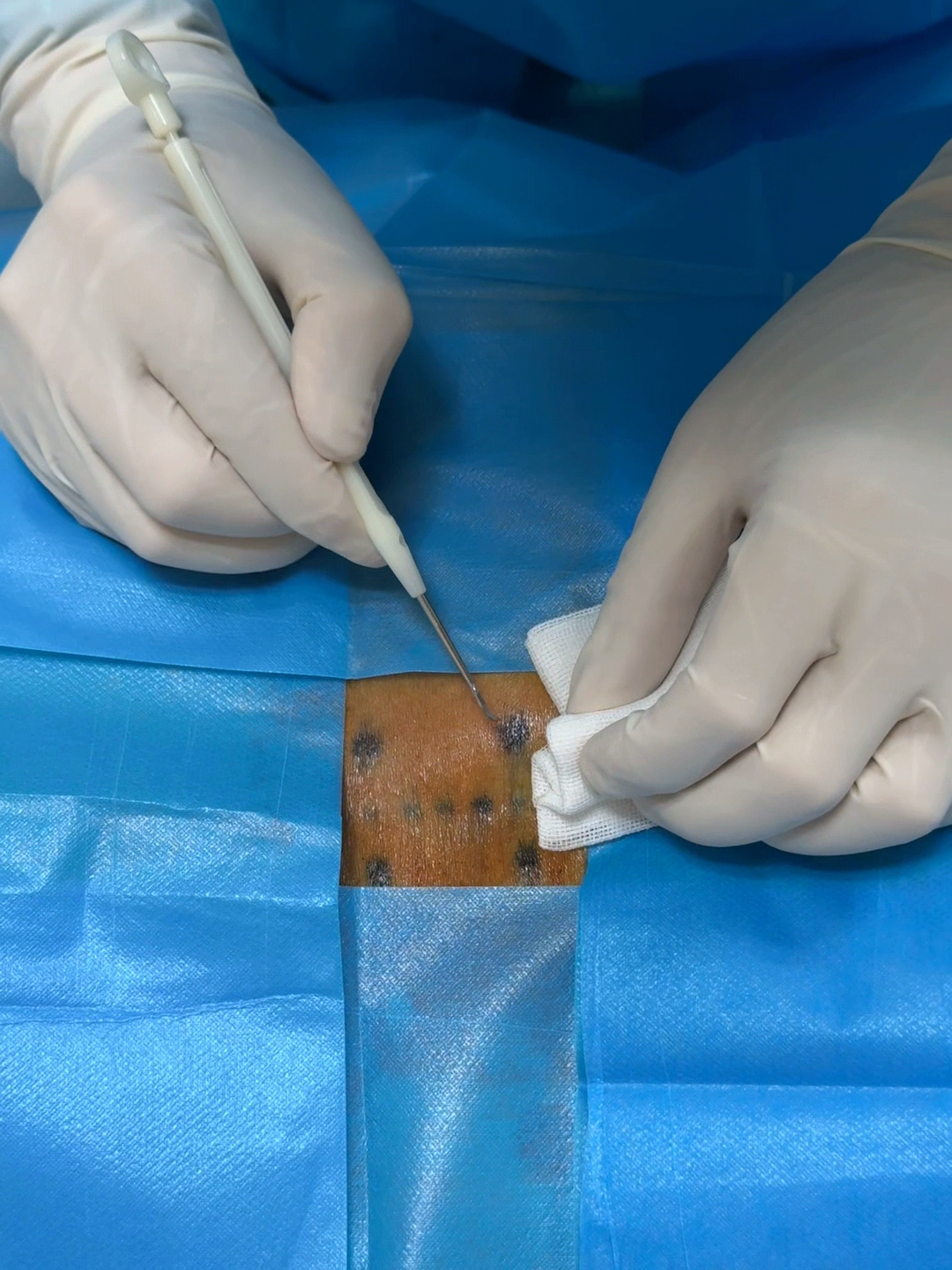

Supplement: Supplementary file 1 — Video S1: Demonstration of the paraspinal hook‐needle intervention. The video demonstrates the controlled insertion, lifting, and releasing movements of the specialized hook‐shaped needle within the selected paraspinal soft‐tissue region. The video is intended to illustrate the procedural technique and does not indicate direct entry into the intervertebral foramen, spinal canal, epidural space, nerve root, dura mater, or sequestered nucleus pulposus. [file CCR3-14-e73226-s001.zip › ccr373226-sup-0002-Supinfo2@The place holder image for video.jpg]
